# Noradrenergic Modulation of Learned and Innate Behaviors in Dopamine Transporter Knockout Rats by Guanfacine

**DOI:** 10.3390/biomedicines11010222

**Published:** 2023-01-15

**Authors:** Anna Volnova, Natalia Kurzina, Anastasia Belskaya, Arina Gromova, Arseniy Pelevin, Maria Ptukha, Zoia Fesenko, Alla Ignashchenkova, Raul R. Gainetdinov

**Affiliations:** 1 Institute of Translational Biomedicine, Saint Petersburg State University, Saint Petersburg 199034, Russia; 2 Biological Faculty, Saint Petersburg State University, Saint Petersburg 199034, Russia; 3 Nevsky Center of Scientific Collaboration, Saint Petersburg 192119, Russia; 4 Saint Petersburg University Hospital, Saint Petersburg 199034, Russia

**Keywords:** dopamine (DA), norepinephrine (NE), dopamine transporter knockout (DAT-KO) rats, ADHD model, guanfacine (GF), spatial working memory, attention

## Abstract

Investigation of the precise mechanisms of attention deficit and hyperactivity disorder (ADHD) and other dopamine-associated conditions is crucial for the development of new treatment approaches. In this study, we assessed the effects of repeated and acute administration of α2A-adrenoceptor agonist guanfacine on innate and learned forms of behavior of dopamine transporter knockout (DAT-KO) rats to evaluate the possible noradrenergic modulation of behavioral deficits. DAT-KO and wild type rats were trained in the Hebb–Williams maze to perform spatial working memory tasks. Innate behavior was evaluated via pre pulse inhibition (PPI). Brain activity of the prefrontal cortex and the striatum was assessed. Repeated administration of GF improved the spatial working memory task fulfillment and PPI in DAT-KO rats, and led to specific changes in the power spectra and coherence of brain activity. Our data indicate that both repeated and acute treatment with a non-stimulant noradrenergic drug lead to improvements in the behavior of DAT-KO rats. This study further supports the role of the intricate balance of norepinephrine and dopamine in the regulation of attention. The observed compensatory effect of guanfacine on the behavior of hyperdopaminergic rats may be used in the development of combined treatments to support the dopamine–norepinephrine balance.

## 1. Introduction

Dysfunction of dopamine regulation leads to numerous neuropsychiatric disorders, such as Parkinson’s disease, Huntington’s disease, schizophrenia and depression, as well as attention deficit hyperactivity disorder (ADHD) [1,2,3,4]. ADHD is a neurodevelopmental disorder characterized by abnormalities in behavior, core features of which include hyperactivity, impulsivity and inattention [2,5,6,7,8]. Studies utilizing animal models are crucial for the understanding of the mechanisms underlying ADHD and for developing new approaches to the treatment of this disorder [9,10,11,12,13,14,15].

Dopamine transporter knockout (DAT-KO) rats, lacking the dopamine transporter gene, demonstrate elevated extracellular dopamine levels in the basal ganglia, a pronounced level of spontaneous hyperactivity and remarkable stereotypical patterns of locomotor activity. It is known that dopamine is involved in various cognitive processes, such as learning, memory and attention, as well as social interactions, goal-directed behaviors and motivation [16,17,18,19,20,21]. DAT-KO rats have impaired working memory, which affects object recognition tasks and conditioned–unconditioned stimulus association learning tasks [9,11,22,23,24]. DAT-KO rats are considered to be a valuable model for investigating putative neurophysiological mechanisms of ADHD, as well as other dopamine-associated pathologies. Due to pronounced hyperdopaminergia, the DAT-KO rat model is particularly promising to study the interactions of the dopaminergic and noradrenergic systems in the regulation of cognitive behaviors and other parameters [24].

It is known that patients with prefrontal DA hypofunction and striatal DA hyperfunction have difficulty differentiating the significance of the stimulus presented [25]. Dopamine and norepinephrine terminals coexist in the prefrontal cortex (PFC) [26] and their interactions seem to be crucial for controlling complex behaviors and, consequently, ADHD symptoms [27,28,29,30,31,32,33].

Innate forms of behavior also depend on the DA and NE balance. Pre-pulse inhibition (PPI), which is a measure of sensorimotor gating, is reduced in patients with such neuropsychiatric disorders as schizophrenia and autism, as well as ADHD, and may serve as a biomarker of these diseases. PPI, therefore, is invaluable for translational research in neuropsychiatry, since it can also easily be assessed in rodents [34,35,36,37,38,39,40].

In this study, we compare the effects of acute and repeated administration of α2A-adrenoceptor agonist guanfacine (GF) on learned and innate behaviors, as well as on the electrophysiological activity of the prefrontal cortex (PFC) and the striatum in DAT-KO rats. By means of adrenergic modulation, GF may have a compensatory effect on the DA imbalance seen in DAT-KO, since DA and NE balance in the PFC provides for its optimal functioning. We hypothesize that repeated GF administration may further improve spatial working memory task fulfillment in the Hebb–Williams maze in DAT-KO rats, because in our previous research on the acute GF administration led to an improvement in that aspect [41]. It is well established that GF is important for improving impairments of memory and attention processes through the modulation of PFC activity [27,42]. To evaluate possible biomarkers and correlates of behavioral changes, we also assessed the plausible effect of GF on brain activity and involuntary attention. Since guanfacine is widely used in long-term pharmacotherapy of ADHD, we aimed to evaluate the possible effects of acute and repeated GF on behavioral and neurophysiological deficits seen in DAT-KO rats.

## 2. Materials and Methods

### 2.1. Animals and Their Maintenance

30 DAT-KO and 30 wild-type (WT) littermate rats, males of the same age (3–4 months), were used in the experiments. During the experiments, the requirements of FELASA in RusLASA for studies using laboratory animals were implemented. The animal study protocol was approved by the Ethics Committee for Animal Research of Saint Petersburg State University, St. Petersburg, Russia No. 131-03-10 of 22 November 2021. Prior to the experiments, the rats were maintained in IVC cages (RAIR IsoSystem World Cage 500; Lab Products, Inc., Seaford, DE, USA) with free access to food and water, at a temperature of 22 ± 1 °C, 50–70% relative humidity and a 12 h light/dark cycle (light from 9 am.). Experiments were carried out between 2 pm. and 6 pm.

### 2.2. Hebb–Williams Maze

#### 2.2.1. Apparatus

**Figure 1:**
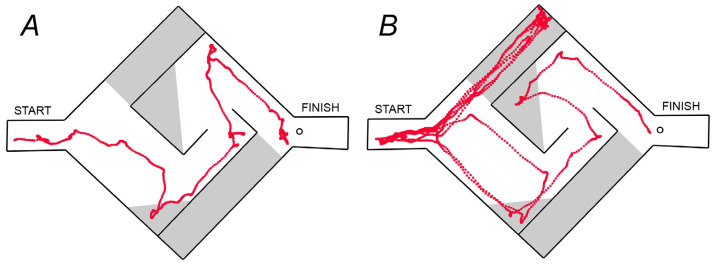
Samples of the visual tracks in the WT rats (**A**) and DAT-KO rats (**B**) in the Hebb–Williams maze. Error zones are indicated in grey color, the circle—the place of food reward.

We used the Hebb–Williams maze to study animal spatial working memory [43,44]. The behavioral task entailed finding the path from start to finish to obtain food reinforcement (Figure 1). The detailed description of the maze and experimental procedure can be found in our previous work [41]. We compared data after the acute GF administration with the results obtained in this study after repeated GF administration. The data obtained after saline, acute (aGF) and repeated (rGF) guanfacine administration were compared.

#### 2.2.2. Experimental Setup

For five days before training, the rats received food at a ratio of 90% of their regular diet (BioPro, Novosibirsk, Russia). Each animal was weighed daily prior to the experiment and throughout all of the experiments’ duration. At the beginning of the experiment, all rats were familiarized with a wall-less maze for 2 days. Then, the rats were trained in the unchanging maze arena for 3 days (three trials for each animal per day) with a reward only in the goal box food well for habituation to the maze and task rules acquisition (for details, see [41]). Then, all animals were divided into groups and then trained for two consecutive days in a new maze arena configuration. Ten WT and 10 DAT-KO rats received repeated GF (rGF, Sigma, St. Louis, MO, USA; daily administration 0.25 mg/kg, i.p.) administration for two weeks leading up to the start of the experiments, and before all experimental sessions, 60 min before testing. Ten other WT and 10 DAT-KO rats were trained in the same way, but they received only the saline administration (0.3 mL, i.p.). The results of the experiments were compared with acute administration of GF (aGF, Sigma, USA; 0.25 mg/kg, i.p., 60 min before testing).

During the experiment, the following behavioral variables were recorded: distance traveled; time to reach the goal; time spent in error zones; the number of return runs. The analysis was performed using a video camera mounted above the maze and video tracking software EthoVision XT11.5 (Noldus Information Technology, Leesburg, VA, USA) (Figure 1).

### 2.3. Acoustic Startle Reaction (ASR) and the Pre-Pulse Inhibition (PPI)

Twenty DAT-KO and 20 wild-type (WT) rats were used in the experiments: 10 WT and 10 DAT-KO rats received saline administration and then (after 4–5 days) acute GF administration (0.25 mg/kg i.p., 60 min before testing), 10 other WT and 10 DAT-KO rats received repeated GF administration (daily, 0.25 mg/kg i.p., 60 min before testing, two weeks prior to the start of the experiments and during all the experimental sessions. The results after saline, acute and repeated administration of GF were compared.

#### 2.3.1. Apparatus

A sensitive platform was used to measure the rat’s startle response; the movement of the animal was detected via four floor-mounted vibration sensors. Force detected by the sensors was converted to voltage analog signals that were digitized and stored. Two high-frequency loudspeakers on the opposite sides of the experimental chamber, mounted above the platform, generated broadband background noise and acoustic stimuli, which were controlled by the data acquisition interface (CED Power1401-3A, Cambridge Electronic Design, Cambridge, UK) and Spike2 software. Sound and accelerometer sensitivity was routinely calibrated (DT-8820, CEM, Shenzhen, China).

#### 2.3.2. Experimental Setup

Prior to the start of a series of experiments, each rat was placed on the platform and exposed to “white noise” with an intensity of 74 dB for 20 min for habituation to the experimental conditions. On the day of the experiment, each animal was presented with “white noise” with an intensity of 74 dB for 10 min, followed by 10 sound stimuli with an intensity of 78 dB and a duration of 50 ms (pre-pulse), which did not result in any motor reactions. Then, 20 acoustic stimuli with an intensity of 100 dB and a duration of 50 ms (pulse) followed, causing a pronounced ASR. Then, 20 combinations of acoustic stimuli (pre-pulse + pulse) followed to register the changes in the ASR amplitude and calculate the pre-pulse inhibition. The interval between pre-pulse and pulse stimuli was 100 ms. The interval between presentations of stimuli or paired stimuli varied from 10 to 14 s to avoid the animal’s habituation to sounds. The trial presentation was controlled by a custom script for Spike2 software (CED, UK). PPI was calculated as a percentage score: ((startle response for pulse alone—startle response for pulse with pre-pulse)/startle response for pulse alone) × 100. The ASR amplitude was recorded by the Spike2 program using a data acquisition interface (CED Power1401, Cambridge Electronic Design, Cambridge, UK) synchronized with the sound stimulus delivery system. During the experiments, we encountered difficulties recording the DAT-KO rats’ reactions, which are characterized by hyperactivity and an increased level of motor activity. In order to distinguish between the ASR and background motor reactions typical of DAT-KO rats, a video camera was synchronized with the recording of the motor reactions and the onset of the acoustic signal presentations. The synchronization allowed to avoid the overlapping of recorded motor responses with traveling around the platform, grooming, and rearings.

### 2.4. LFP Power Spectra and Coherence

The local field potential (LFP) recordings were carried out in 25 adult male rats: WT (n = 12) and DAT-KO (n = 13). Six WT and six DAT-KO rats received saline administration. Then, after 4–5 days, these animals received an acute administration of GF (0.25 mg/kg, i.p., 60 min before testing). Six WT and seven DAT-KO rats received repeated GF (daily administration, 0.25 mg/kg i.p., 60 min before testing) for two weeks leading up to the start of the experiments.

#### 2.4.1. Surgical Procedures

Electrodes for brain activity recordings were mounted on the skull under isofluranum anesthesia with the use of a micromanipulator in a stereotaxic frame. Three electrodes were implanted into each animal. An epidural screw (1 mm in diameter; 1 mm in length; steel) was used as a reference electrode (AP= −7 mm; L= 3 mm, coordinates relative to bregma). Two intracerebral electrodes (50 μm in diameter; 2.5 mm/5 mm in length; tungsten wire in perfluoroalkoxy polymer isolation) were used for the LFP recordings. We used the following coordinates relative to bregma: prefrontal cortex (PFC) intracerebral electrode AP = +2 mm; L = 2.5 mm; 2.5 mm in length; and striatal (Str) intracerebral electrode AP = 0 mm; L = 3 mm; 5 mm in length.

#### 2.4.2. EEG and LFP Recordings

The experiments were carried out 2–3 days after surgery. The experimental setting for LFP recordings consisted of an amplifier (×1000 gain), Cambridge Electronic Design (CED) Power1401-3A data acquisition interface, and Spike2 software (CED), sampling rate 25,000 Hz. During the recording process, the animals were placed in a 25 cm × 25 cm × 25 cm Plexiglas box located within a Faraday cage.

Brain activity was recorded in six WT and six DAT-KO rats, on two subsequent days: for one hour (60 min) after a saline injection (0.9% NaCl i.p., 30 min before the recording), and for one hour after an acute GF injection (0.25 mg/kg, i.p., 60 min before the recording). Brain activity after a repeated GF injection was recorded in six WT and seven DAT-KO rats (daily administration, 0.25 mg/kg i.p. for two weeks prior to the start of recording). For behavior monitoring, video was recorded simultaneously with brain activity. Only parts of the recordings where the animals were awake were used in the subsequent analysis.

#### 2.4.3. Analysis of EEG and LFP Activity

For each recording, 200 s without artifacts were selected for analysis. The sampling rate of the recordings was 1000 Hz. The data were analyzed with a script (COHER.s2s, CED official website), which calculates the power spectra of a signal through fast Fourier transform and the coherence of two signals. A power spectrum is the ratio of frequency to power. Coherence values are between 0 and 1, and it is equal to 1 for a particular frequency if the phase shift between the waveforms is constant, and the amplitudes of the waves have a constant ratio. Data in the 0–0.8 range were excluded due to the abundance of artifacts. The following ranges for the electroencephalographic rhythms were used for analysis and interpretation (in Hz): delta (0.9–3), theta (4–8), alpha (9–11), lower beta (12–19), higher beta (20–29), lower gamma (30–48), higher gamma (52–74).

### 2.5. Statistical Analysis

The data were presented as the mean ± SEM; *p* < 0.05 was considered as statistically significant for all tests. A preliminary assessment of the normality of the data distribution was performed using the Kolmogorov–Smirnov test. To analyze the parameters of the rats’ Hebb–Williams maze behavior and pre-pulse inhibition, we used a two-way analysis of variance (two-way ANOVA, genotype factor: WT or DAT-KO; treatment factor: saline, acute and repeated guanfacine administration) with a post-hoc Sidak multiple comparison test (WT vs. DAT-KO; saline vs. aGF; saline vs. rGF; saline vs. aGF). To analyze the power spectra density of the local field potential (LFP) from the striatum and prefrontal cortex, and Str-PFC coherence, we used an ordinary one-way ANOVA with post-test for linear trend or Sidak’s multiple comparisons post-hoc test (for parametric data) and Kruskal–Wallis test with Dunn’s multiple comparisons test (for nonparametric data).

## 3. Results

### 3.1. Hebb–Williams Maze

**Figure 2:**
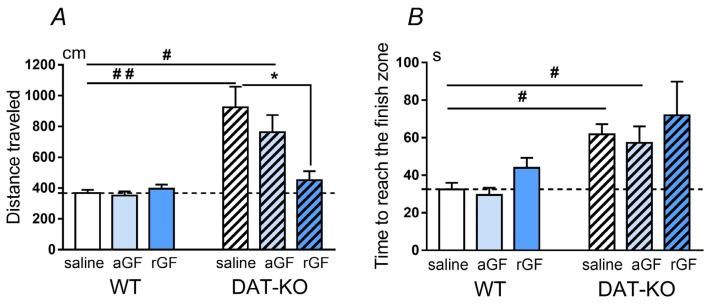
Comparison of the distance traveled (in cm), (**A**) and the time for reaching the goal box (in s), (**B**) of the Hebb–Williams maze in DAT-KO and WT rats after saline, acute (aGF) and repeated (rGF) guanfacine administration. Results are presented as the mean ± SEM, # *p* < 0.05; ## *p* < 0.01, Wilcoxon signed rank test; * *p* < 0.05; two-way ANOVA test combined with Sidak’s multiple comparisons post-hoc test.

The analysis of the motor behavior of DAT-KO rats in the Hebb–Williams maze showed pronounced hyperactivity while performing behavioral tasks, in comparison to the WT rats [41]. We observed that the DAT-KO rats traveled significantly longer distances (*p* < 0.01) and required more time to reach the goal box (*p* < 0.001), in comparison with the WT rats (Figure 2A,B). Acute GF administration did not induce any significant changes in these behavioral parameters in WT rats. A comparative analysis of the behavioral parameters of WT and DAT-KO rats showed that following acute GF administration, the level of all behavioral parameters was also higher in DAT-KO, in comparison to WT rats after saline (*p* < 0.05 for the distance traveled and for the time to reach the finish zone). However, repeated GF administration resulted in a decrease in the distance traveled by the DAT-KO rats down to levels observed in the WT counterparts (Figure 2A). The time that DAT-KO rats spent in the arena before the rats reached the goal box, in comparison to that of the WT controls (Figure 2B), was longer after saline and acute GF administration (*p* < 0.05), but not after repeated GF administration. 

The two-way ANOVA analysis revealed a significant difference in the distance traveled (genotype factor, *p* < 0.05, treatment factor *p* < 0.0001) and in the time of reaching the goal box (treatment factor *p* < 0.001) between the two groups of rats (Table S1 of Supplementary Materials and Table S2 of Supplementary Materials)

**Figure 3:**
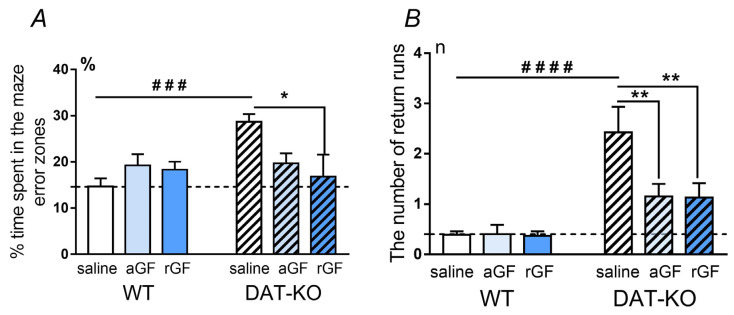
Comparison of the percentage of time spent in the Hebb–Williams maze error zones (in %), (**A**) and the number of return runs (**B**) by the DAT-KO and WT rats after saline, acute (aGF), and repeated (rGF) guanfacine administration. Results are presented as the mean ± SEM; ### *p* < 0.001, #### *p* < 0.0001; * *p* < 0.05, ** *p* < 0.01, two-way ANOVA test combined with Sidak’s multiple comparisons post-hoc test.

The amount of time the animal spent in the error zones (Figure 3A) was an indicator of the test performance: according to this indicator, the spatial navigation in the DAT-KO rats was worse than in the WT rats (## *p* < 0.01). In contrast to saline administration, acute and repeated GF administration decreased the time spent in the error zones approaching the parameters of the WT rats, resulting in no statistical differences from the WT rats after saline administration (Figure 3A, Table S3).

The number of stereotypic (perseverative) reactions was recorded on the basis of the number of returns to the start zone of the maze (Figure 3B). Analysis of this parameter showed that the perseverative activity after saline administration in DAT-KO rats was significantly higher than in WT rats (*p* < 0.001, two-way ANOVA with Sidak’s multiple comparisons post-hoc test). The acute and repeated GF administration lead to a decrease in the number of returns, in comparison to saline administration (*p* < 0.01; two-way ANOVA with Sidak’s multiple comparisons post hoc test; Table S4 of Supplementary Materials) down to levels not reaching a significant difference from the control values for the WT rats.

The data obtained indicate that the prolonged period of the GF administration may further improve the spatial task performance. It is apparent that a longer period of GF treatment makes the task performance more effective, due to a significant decrease in the time spent in the error zones and the distance traveled. These findings suggest that GF may improve attention processes. A decrease in the stereotypical (perseverative) activity patterns of mutants was also observed after acute and repeated GF administration.

### 3.2. Acoustic Startle Reaction (ASR) and the Pre-Pulse Inhibition (PPI)

**Figure 4:**
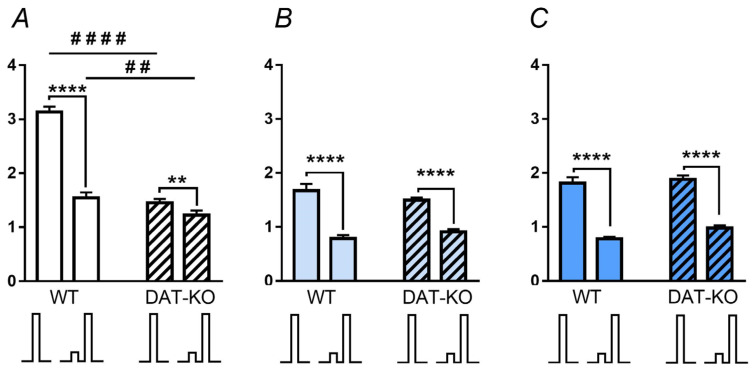
Comparison of the amplitude of the acoustic startle reaction (ASR, in mV) in DAT-KO and WT rats after saline (**A**), acute (**B**) and repeated (**C**) guanfacine administration. Results are presented as the mean ± SEM; ## *p* < 0.01, #### *p* < 0.0001, ** *p* < 0.01, **** *p* < 0.0001 according the Mann–Whitney test.

The ASR was investigated in the WT and DAT-KO rats after saline, acute, and repeated GF administration, and then the PPI values were calculated. In the WT rats after saline administration, ASR on the paired (pulse and pre-pulse) presentation was lower than the responses evoked by the pulse alone (Figure 4A). DAT-KO rats showed a similar trend, but the ASR amplitudes were lower than in the WT rats. In contrast to saline, the acute and repeated GF administration induced a decreased ASR for double acoustic stimuli (pre-pulse + pulse) in both WT and DAT-KO rats (Figure 4A–C).

**Figure 5:**
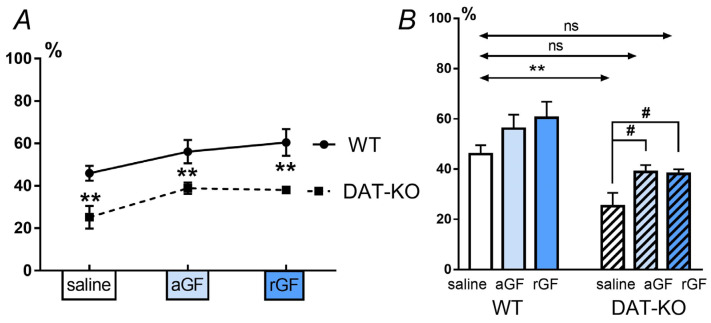
Pre-pulse inhibition (PPI) in DAT-KO and WT rats after saline, acute (aGF) and repeated (rGF) guanfacine administration; comparison of the groups of animals of different genotypes (**A**) and comparison of the effect of the administered drugs (**B**). Results are presented as the mean ± SEM; ** *p* < 0.01 according to Fisher’s LSD post-test; # *p* < 0.05; ns–(not significant) two-way ANOVA and Dunn’s multiple comparisons post-hoc test.

For detecting the differences between the amplitude of the responses elicited by the single (pulse) and double (pre-pulse + pulse) stimuli, we analyzed the value of the pre-pulse inhibition (PPI). Analysis of the data with a two-way ANOVA showed that for the factor “genotype”, the differences are significant with *p* < 0.0001, while for the factor “treatment” —with *p* < 0.01, no interaction between the genotype and treatment was observed. Comparison of the results in DAT-KO and WT rats showed that after saline administration, the DAT-KO rats showed less a pronounced PPI than the WT rats (Figure 5A). Multiple comparisons between the mean values for the WT and DAT-KO (Fisher’s least significant difference) showed significant differences between groups for each drug (*p* < 0.01) (Figure 5A, Table S5).

A comparison of the PPI index values for WT and DAT-KO rats after saline administration, showed significant differences (Figure 5B). These data show that the WT rats have normal processes of habitation and sensorimotor gating, while in the DAT-KO rats, the PPI index is reduced, indicating the impaired sensory information perception. In contrast to this, the GF administration (both acute and repeated) led to an increase in the PPI index in the knockout animals, and it became similar to that in the WT after saline (Figure 5B). An increase in the PPI index in the DAT-KO rats after acute and repeated administration of GF indicates an improvement in the sensorimotor gating.

### 3.3. Power Spectra and Coherence of the Brain Activity

To analyze the power spectra of the neural activity in the prefrontal cortex (PFC) and dorsal striatum (Str), we used a traditional LFP analysis, based on the signal decomposition into simpler sinusoidal harmonics. We compare electroencephalographic rhythms according to the following ranges (in Hz): delta (0.9–3), theta (4–8), alpha (9–11), lower beta (12–19), higher beta (20–29), lower gamma (30–48), higher gamma (52–74).

The differences between the power spectra and intrinsic coherence between PFC and Str in DAT-KO and WT rats after saline administration, were assessed in our previous article [45]. In the present research, we focused on the effects of acute and repeated guanfacine administration on these parameters.

**Figure 6:**
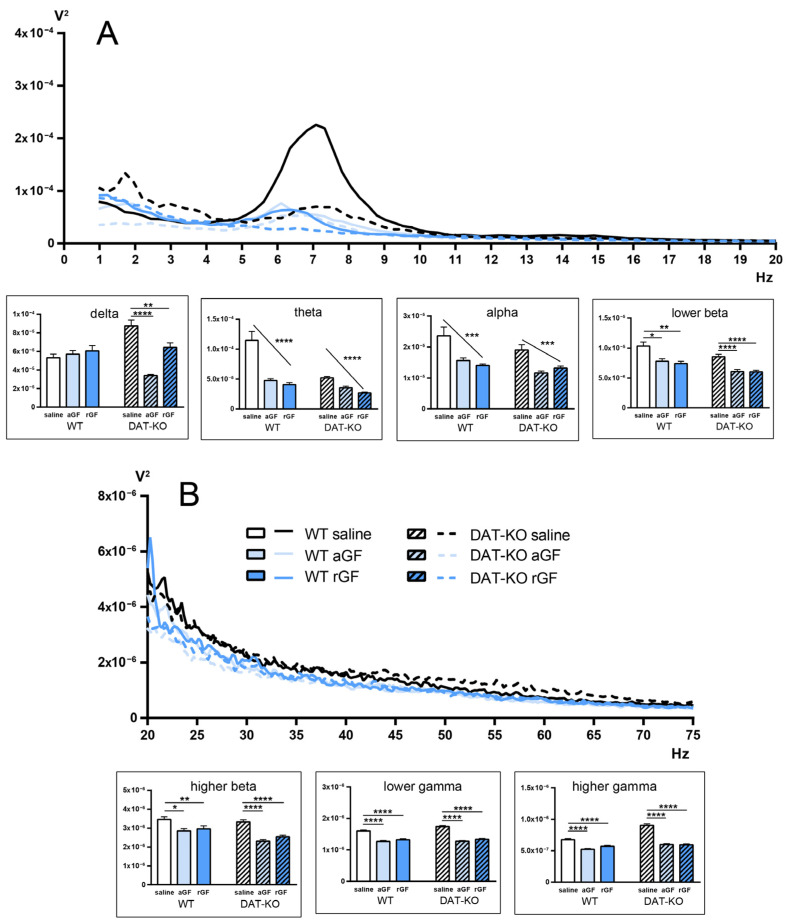
Power spectra of the brain activity in DAT-KO and WT rats recorded from the striatum (Str) after saline, acute (aGF) and repeated (rGF) guanfacine administration; (**A**) 1–20 Hz range, (**B**) 20–75 Hz range. Data are presented as the mean ± SEM. The diagrams represent the following electroencephalographic rhythms (in Hz): delta (0.9–3), theta (4–8), alpha (9–11), lower beta (12–19), higher beta (20–29), lower gamma (30–48), higher gamma (52–74); one-way ANOVA, * *p* < 0.05; *** *p* < 0.001; **** *p* < 0.0001 post test for the linear trend; ** *p* < 0.01 Dunnett’s multiple comparisons test.

Guanfacine administration to WT and DAT-KO rats led to an overall decrease in the power spectral density in the LFP activity of the dorsal striatum (Figure 6). This fact is confirmed by the presence of a linear trend, according to a 2-way ANOVA (indicated by the sloping line in the diagrams). Note the high reliability of the observed differences (*p* < 0.0001 and *p* < 0.001), as well as the unidirectional changes occurring in the power spectrum in WT and DAT-KO rats in all main bands (Table S6 of Supplementary Materials).

**Figure 7:**
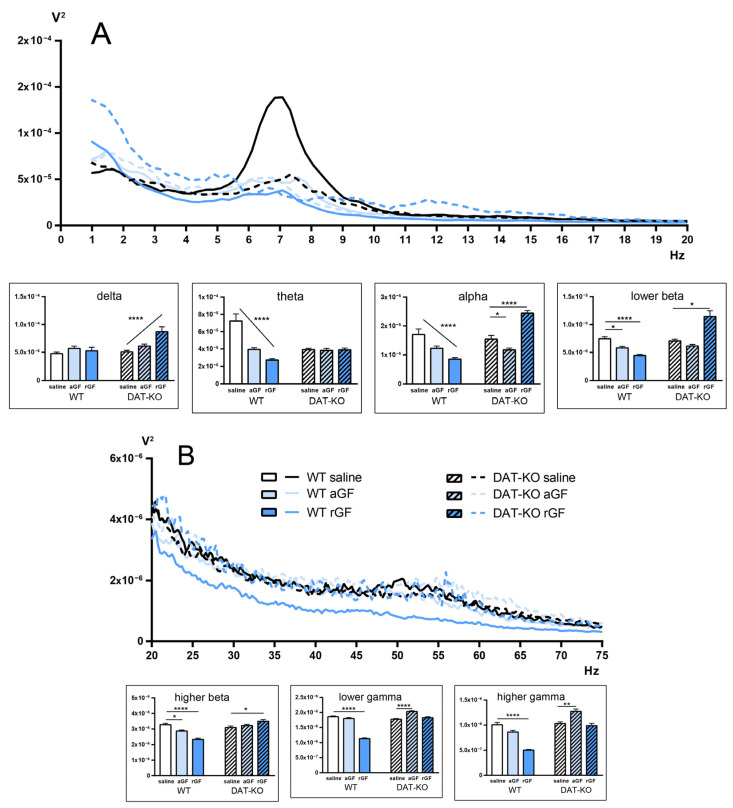
Power spectra of the brain activity of DAT-KO and WT rats recorded from the prefrontal cortex (PFC) after saline, acute (aGF) and repeated (rGF) guanfacine administration; (**A**) 1–20 Hz range, (**B)** 20–75 Hz range. Data are presented as mean ± SEM. The diagrams represent the following electroencephalographic rhythms (in Hz): delta (0.9–3), theta (4–8), alpha (9–11), lower beta (12–19), higher beta (20–29), lower gamma (30–48), higher gamma (52–74); one-way ANOVA, **** *p* < 0.0001 post test for the linear trend; * *p* < 0.05; ** *p* < 0.01 Dunnett’s multiple comparisons test.

Analysis of the activity recorded in the PFC showed different tendencies (Figure 7; Table S7 of Supplementary Materials) in the two groups of rats. In WT rats, the aGF and rGF administration resulted in a significant decrease in the power spectra of the PFC activity in theta, alpha and lower beta ranges. On the contrary, in the high frequency areas, the power spectral density increased in the higher beta and gamma ranges. No changes were observed in the delta range in WT rats.

In DAT-KO rats, administration of aGF and rGF generally led to an increase in the power spectra of the PFC activity, except for the theta range. We found that this increase was unstable at different ranges. It can be concluded that aGF and rGF administration might lead to the changes in the frequency-time characteristics of LFP in DAT-KO rats, similarly to those in WT rats.

**Figure 8:**
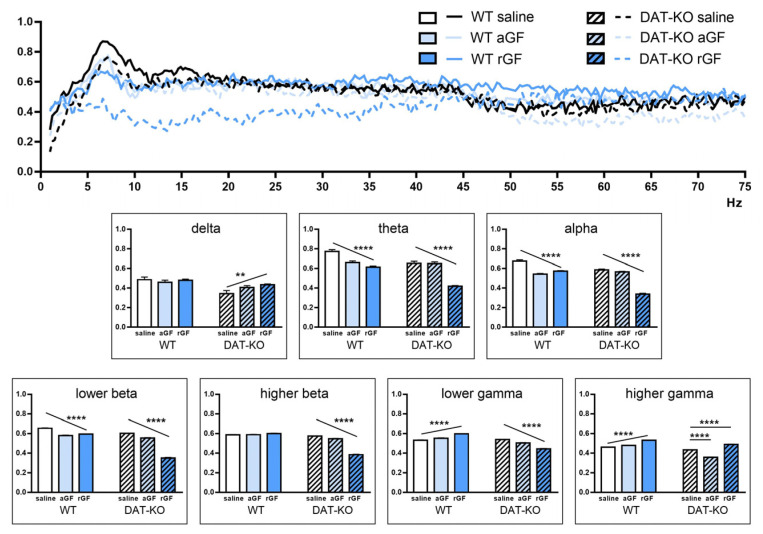
Coherence of the brain activity in PFC and Str in DAT-KO and WT rats after saline, acute (aGF) and repeated (rGF) guanfacine administration. Degree of coherence is expressed in fractions of one. Data are presented as the mean ± SEM. The diagrams represent the following electroencephalographic rhythms (in Hz): delta (0.9–3), theta (4–8), alpha (9–11), lower beta (12–19), higher beta (20–29), lower gamma (30–48), higher gamma (52–74); two-way ANOVA, ** *p* < 0.01 **** *p* < 0.0001 post test for the linear trend and Dunnett’s multiple comparisons test.

It was shown earlier that the DAT-KO rats are characterized by lower values of the coherence coefficient between the investigated brain regions (Str and PFC). In WT rats, we observed a decrease in coherence at lower frequencies and its increase in the gamma range (Figure 8; Table S8). In DAT-KO rats, a decrease in coherence was observed in all ranges except the lowest and highest. The delta range differed from the others in the character of the observed changes in both WT and DAT-KO rats. We assume that our findings might be associated with a greater variability of the values in this frequency range.

## 4. Discussion

Attention deficit hyperactivity disorder is a common psychiatric disease in childhood. Over 50% of children with this disorder continue to exhibit its symptoms into adulthood [46,47]. ADHD is characterized by a high comorbidity with other developmental disorders, anxiety and mood disorders, tics, learning disabilities and sleep disorders [48,49,50,51]. Numerous studies underline the importance of normal brain development for the absence of neuropsychiatric symptoms [15]. Moreover, multiple factors contribute to the emergence of ADHD symptoms, including abnormalities in neurotransmission, structural changes in various brain structures, altered functional connectivity, stress, inflammation, etc. [48,49,52,53,54]. The precise mechanisms underlying ADHD symptoms are still poorly understood, thus investigation of pathophysiological states of the relevant brain networks in animal models becomes increasingly relevant. DAT-KO rats, lacking the dopamine transporter gene, mimic the main endophenotypes of ADHD patients, including hyperactivity, impulsivity and inattention. Behavioral changes seen in DAT-KO rats are, at least in part, due to extremely elevated levels of extracellular dopamine in the striatum [11], caused by the absence of DAT. Despite their pronounced hyperactivity, DAT-KO rats are able to learn a behavioral task in the 8-arm radial maze [22], and object recognition task in a RedBox paradigm [23], although with a poorer performance, in comparison to WT animals. It should be noted that, while learning cognitive tasks, hyperdopaminergic rats may use unique behavioral tactics [22]. In contrast to WT rats, DAT-KO rats show numerous perseverative reactions, which lead to an increased number of erroneous trials during the learned task performance [23].

In this study, we evaluated the changes in innate and learned behaviors of DAT-KO rats under the long-term treatment with guanfacine, a α2A-adrenoceptor agonist, which is widely used in ADHD treatment. In our experiments, repeated GF injections ameliorated the task fulfillment and PPI in DAT-KO rats. The distance traveled decreased, the time of reaching the goal box increased in mutants, while a decrease in the time spent in the erroneous zones was observed. The GF administration also reduced the number of perseverative reactions. Comparable tendencies were described in our previous work with acute administration of GF [41]. In contrast to WT rats, the DAT-KO rats demonstrated an improvement in the task fulfillment in the Hebb–Williams maze after repeated GF. These results might be indicative of improved attention processes in DAT-KO rats following GF administration.

There are numerous studies supporting the key role of DAT in the development of several dopamine related pathologies [55,56,57,58,59]. It is not surprising, since dopamine dynamics in dopaminergic terminals and synapses are, to a large extent, regulated by DAT. Since DA has a high affinity for NET, in NET-enriched areas, such as the PFC, NET regulates dopamine, as well as the norepinephrine levels. Thus, drugs affecting DAT or NET can regulate dopamine storage and release by noradrenergic neurons and are important for ADHD treatments [60].

It is well known that dopamine and norepinephrine terminals project to the prefrontal cortex (PFC) [61,62,63]. Dysfunction of the DA pathways leads to a lack of attention and to intolerance in waiting for a reward in patients with ADHD [64]. NE pathways from the locus coeruleus (LC) are involved in attention control. In ADHD patients, discharges from the LC are altered, thus complicating focused attention [65]. Dendritic spines on pyramidal neurons in the PFC have α2A adrenergic receptors and D1 dopamine receptors involved in PFC functions. NE has high affinity for the postsynaptic α2A adrenergic receptors and enhances cognitive functions. Normal PFC functioning requires optimal levels of catecholamines. A catecholamine deficiency leads to a reduced level of control of hyperactivity and attention deficit and the poor planning of goal-directed behavior [66]. Projections from the PFC to the Str constitute a part of the network that ensures the functioning of the working memory. The PFC-Str activities appear to contribute to a correct action after a period of working memory consolidation, resulting in the successful completion of the working memory task [67]. Moreover, it has been suggested that the PFC-Str projection pathways are selectively involved in inhibitory control [68]. It is also important to note that an individual‘s emotional state is strongly connected to a proper performance in a task. DA and NE are known to participate in many basic emotions. PFC is one of the main structures that controls cognitive functions and motivation-based behaviors. There is an opinion that DA induces happiness and pleasure, whereas NE is related to fear and anger states [69]. The control of emotional states may facilitate the interruption of ongoing behaviors, such as impulsive behavior, which is one of the core features of ADHD [70,71].

There are several approaches to ADHD treatment, including pharmacological interventions [72,73]. Psychostimulants, including amphetamine and methylphenidate, are most commonly used [74,75]. Non-psychostimulant drugs, such as guanfacine (α2A-adrenoceptor agonists) and atomoxetine (norepinephrine transporter inhibitor) are also used for counteracting ADHD symptoms as monotherapy and add-on therapy [73,76]. The action mechanism of NE-based treatments has been partially investigated in animal models. The acute and chronic administration of NE-based anti-ADHD drugs has been shown to selectively activate the prefrontal catecholamine systems in mice [77], while the systemic administration of the a2A-adrenoceptor agonist GF reduced the impulsive choice behavior in rats [78]. It has been shown that in juvenile SHR rats tested in a five-trial inhibitory avoidance task, the stimulation of postsynaptic α2A adrenergic receptors by GF leads to attention improvement [79]. There is an opinion that GF affects the dendritic branching of the pyramidal cells in the PFC, thus improving the cognitive performance [80]. There are also other data indicating that GF enhances the PFC regulation of attention and improves the performance in working memory tasks [14,78,81,82]. Thus, GF administration in DAT-KO rats induces neuropharmacological effects that may explain the results of the behavioral tests. The beneficial effects of GF are associated with an improved noradrenergic modulation in the neuronal circuits which involve the PFC [83]. It has been proposed that guanfacine is most specific for the treatment of the prefrontal attentional and working memory deficits [27,42].

Such innate behaviors as the acoustic startle reaction and pre-pulse inhibition (PPI) are linked to the habituation and ability to rapidly adjust to a changing environment. These innate forms of behavior may also depend on the DA-NE balance. It was described that the PPI, which is a measure of sensorimotor gating, is reduced in patients with several neuropsychiatric disorders, such as schizophrenia, autism and ADHD. PPI findings in humans and rodents show its importance for the translational investigation in neuropsychiatry [35,36,37,39,40]. Habituation to a new environment is deficient in DAT-KO rats [84,85]. DAT-KO mice and rats display a consistent PPI deficit [11,86]. The enhancement of cortical extracellular DA via the blockade of NET, leads to a reversal of this deficit in DAT-KO mice [87], as well as in rats [45]. It was also shown that the depletion of the prefrontal DA induces a PPI deficit in rats [88]. At the same time, it is known that dopaminergic agonists increase the ASR amplitude and shorten its latency [89]. Our findings showed that a PPI deficit in hyperdopaminergic rats is improved by both acute and repeated GF administration, thus validating an important role of the NE and DA interaction in the regulation of this process.

These observations show that activation of the alpha-2A-adrenoceptors ameliorate both learned and innate forms of behavior. In our previous work [45] we suggested that the possible NE-mediated activation of the PFC (produced by either adrenoceptor activation or NET-inhibition) seems to have a positive effect on attention and perseverative reactions, but not hyperactivity, which could be mainly regulated by the DA in the PFC. The results of the current study might challenge that assumption, since prolonged guanfacine administration significantly decreased the distance traveled by DAT-KO animals before reaching the goal box, making this parameter indistinguishable from that of the WT rats. This result may be interpreted as a correlate of reduced hyperactivity.

One of the significant neurophysiological correlates of ADHD is an increase in the power of the electrophysiological activity in the theta band [90]. However, as was described in detail in our previous study [45], a lower level of theta band power in knockout animals is observed both in the striatum and in the PFC. DAT-KO rats are also characterized by lower values of coherence in the investigated brain regions (Str and PFC). In this study, we attempted to evaluate the effects of acute and repeated administration of guanfacine on the electrophysiological characteristics of brain activity in DAT-KO rats to assess the possible biomarkers of behavioral changes. In WT rats, we observed a decrease in coherence at lower frequencies and its increase in the gamma range under GF. In DAT-KO rats under GF, a decrease in coherence was observed in almost all ranges, except the lowest and highest. The delta range differed from the others in the character of the observed changes in both WT and DAT-KO rats. We assume that these findings might be associated with a greater variability of values in this frequency range.

However, DAT-KO rats under GF showed a reduced activity in the alpha-beta bands consistent with the data reported for patients with ADHD [91,92]. An increase in power in the alpha range, which is ameliorated by GF, correlates with a slower reaction time, higher reaction time variability, and an overall tendency towards a lower performance in tasks measuring inhibition in children with ADHD [91].

It has been proposed that the GF action on α2A-adrenoceptors leads to a more effective regulation of attention and goal-directed behaviors by the PFC and to the strengthening of its functional connections with other cortical areas [93,94]. It was proposed that GF reduces the thalamo-cortical excitability and thereby effectively modulates cognitive processing [42]. In WT rats, GF induces a decrease in low-frequency bands, including the alpha band. Moreover, a decrease in the coherence index of the alpha band and lower frequencies was found. Phase synchronization of alpha bands in different areas of the brain provides effective network communication [95]. Therefore, changes in the coherence index may indicate that functional connectivity within the local brain areas becomes weaker, which is indicated by the decrease in the higher frequencies, while that of large scale areas becomes stronger. It is important to note that the increase in coherence in WT rats in the gamma band under GF indicates its influence on local neuronal ensembles involved in information processing. It means that despite the absence of external changes in the behavioral tests described above, GF stimulates a significant functional reorganization of the neuronal communication in WT rats.

The effect of GF injections on the time-frequency characteristics of neuronal activity in the brain of DAT-KO rats is not as unambiguous. Only acute administration of GF leads to a decrease in the alpha band in the PFC and striatum, whereas repeated administration has opposite effects eliciting a strong power spike. In contrast to acute administration, the cumulative effect of repeated administration of GF results in a stronger decline in the coherence index in the low-frequency range, including the alpha band. Our data are comparable to the findings on the changes in the task-related alpha (8–12 Hz) interhemispheric connectivity correlated with inattentive symptom severity [96].

To sum up, the effects of repeated GF administration on both DAT-KO and WT rats are mostly comparable to those seen after acute administration. In humans, however, both in childhood and adulthood, a gradual improvement in ADHD symptoms, has been reported under long-term GF, seen only after several weeks of treatment [42,97,98]. In our study, a similar tendency can be seen in locomotor activity in the Hebb–Williams maze, which improved more significantly under repeated GF than under acute GF and can be seen as a sign of reduced hyperactivity. Some differing changes can also be seen in brain activity, the interpretation of which requires further investigation, however, it may turn out to be correlates of certain behavioral changes.

## 5. Conclusions

In this study, acute and repeated guanfacine (GF), α2A-adrenoceptor agonist, was shown to influence innate and learned behaviors in DAT-KO (dopamine transporter knockout) rats, as well as the electrophysiological correlates of brain activity. The results obtained, combined with our previous studies, show that noradrenergic modulation improves different aspects of behavior in hyperdopaminergic knockout rats. GF administration improved the fulfillment of a learned spatial tasks and ameliorated the PPI in DAT-KO rats. Changes in the electrophysiological activity of the brain under GF proved to be similar to those observed in human patients. Overall, the impact of repeated GF was found to be mostly comparable to the acute administration, except for locomotor activity, which improved further with long-term administration, and some electrophysiological parameters in DAT-KO rats. The results obtained in DAT-KO and WT rats under acute and repeated GF, allow for the development of further hypotheses on differential effects of DA and NE networks on various forms of behavior and, possibly, in different cognitive and psychiatric disorders.

## 6. Limitations and Future Directions

Our study, as preclinical research on an animal model, has limitations, since there is no direct application of the findings to the clinical practice. Moreover, the DAT-KO rats, as an animal model, are supposedly not sufficient for assessing the full range of ADHD symptoms. The future directions of our studies involve the use of other non-psychostimulants in different cognitive tasks, a comparison of different doses of drugs, as well as other, more precise, methods of targeting specific neuronal networks. It is also important to conduct longitudinal experiments, in order to search for doses and combined drug applications for ameliorating ADHD symptoms long-term and understanding their mechanisms.

## Data Availability

The raw data used in this study are available upon request from the corresponding author.

## Supplementary Materials

The following supporting information can be downloaded at: https://www.mdpi.com/article/10.3390/biomedicines11010222/s1, Table S1. The statistical analyses of the distance traveled by DAT-KO and WT rats in Hebb-Williams maze after saline, acute (aGF), and repeated (rGF) guanfacine administration; Table S2. The statistical analyses of the time of reaching the goal box of Hebb-Williams maze by DAT-KO and WT rats after saline, acute (aGF), and repeated (rGF) guanfacine administration. Table S3. The statistical analyses of the percentage of time spent in the Hebb-Williams maze error zones by DAT-KO and WT rats after saline, acute (aGF), and repeated (rGF) guanfacine administration. Table S4. The statistical analyses of the number of return runs by DAT-KO and WT rats in the Hebb-Williams maze after saline, acute (aGF), and repeated (rGF) guanfacine administration. Table S5. The statistical analyses of pre-pulse inhibition index (PPI) for DAT-KO and WT rats after saline, acute (aGF), and repeated (rGF) guanfacine administration. Table S6. The statistical analyses of power spectral density of striatum LFP at various frequency bands in WT and DAT-KO rats. Table S7. The statistical analyses of power spectral density of prefrontal cortex LFP at various frequency bands in WT and DAT-KO rats. Table S8. The coherence between the activity of the prefrontal cortex (PFC) and Striatum (Str) at various frequency bands.
