# Supplementary material for: Noradrenergic Modulation of Learned and Innate Behaviors in Dopamine Transporter Knockout Rats by Guanfacine
Source: Biomedicines. 2023 Jan 15;11(1):222. doi: 10.3390/biomedicines11010222 (PMC9856099; doi:10.3390/biomedicines11010222)
Supplement: Supplementary file 1 [file biomedicines-11-00222-s001.zip › biomedicines-2101737-supplementary.pdf]

Table S1. The statistical analyses of the distance traveled by DAT-KO and WT rats in Hebb-Williams maze after saline, acute (aGF), and repeated (rGF) guanfacine administration. Two-way analysis of variance (two-way ANOVA) with Dunn's multiple comparisons post-hoc test was used. Statistically significant values indicated in green; ns – not significant.

Two-way ANOVA			
Interaction		**	p=0.0071
treatment		****	p<0.0001
genotype		*	p=0.0222
Sidak's multiple comparisons post-hoc test			
	treatment		
WT vs. DAT-KO	saline	****	p<0.0001
WT vs. DAT-KO	aGF	**	p=0.0066
WT vs. DAT-KO	rGF	ns	p>0.9999
	genotype		
saline vs. aGF	WT	ns	p>0.9999
saline vs. rGF	WT	ns	p>0.9999
saline vs. aGF	DAT-KO	ns	p=0.9080
saline vs. rGF	DAT-KO	**	p=0.0017
compared to WT with saline administration			
WT saline vs. DAT-KO saline		****	p<0.0001
WT saline vs. DAT-KO aGF		**	0.0034
WT saline vs. DAT-KO rGF		ns	0.9537

Table S2. The statistical analyses of the time of reaching the goal box of Hebb-Williams maze by DAT-KO and WT rats after saline, acute (aGF), and repeated (rGF) guanfacine administration. Two-way analysis of variance (two-way ANOVA) with Dunn's multiple comparisons post-hoc test was used. Statistically significant values indicated in green; ns – not significant.

Two-way ANOVA			
Interaction		ns	p=0.1145
treatment		***	p=0.0003
genotype		ns	p=0.8223
Sidak's multiple comparisons post-hoc test			
	treatment		
WT vs. DAT-KO	saline	*	p=0.0262
WT vs. DAT-KO	aGF	*	p=0.0441
WT vs. DAT-KO	rGF	ns	p>0.9999
	genotype		
saline vs. aGF	WT	ns	p>0.9999
saline vs. rGF	WT	ns	p=0.9596
saline vs. aGF	DAT-KO	ns	p>0.9999
saline vs. rGF	DAT-KO	ns	p=0.9315
compared to WT with saline administration			
WT saline vs. DAT-KO saline		**	p=0.0070
WT saline vs. DAT-KO aGF		*	p=0.0376
WT saline vs. DAT-KO rGF		ns	p=0.4229

Table S3. The statistical analyses of the percentage of time spent in the Hebb-Williams maze error zones by DAT-KO and WT rats after saline, acute (aGF), and repeated (rGF) guanfacine administration. Two-way analysis of variance (two-way ANOVA) with Dunn's multiple comparisons post-hoc test was used. Statistically significant values indicated in green; ns – not significant.

Two-way ANOVA			
Interaction		**	p=0.0047
treatment		*	p=0.0372
genotype		ns	p=0.2582
Sidak's multiple comparisons post-hoc test			
	treatment		
WT vs. DAT-KO	saline	***	p=0.0007
WT vs. DAT-KO	aGF	ns	p>0.9999
WT vs. DAT-KO	rGF	ns	p>0.9999
	genotype		
saline vs. aGF	WT	ns	p=0.9533
saline vs. rGF	WT	ns	p=0.9941
saline vs. aGF	DAT-KO	ns	p=0.1755
saline vs. rGF	DAT-KO	*	p=0.0268
compared to WT with saline administration			
WT saline vs. DAT-KO saline		***	p=0.0007
WT saline vs. DAT-KO aGF		ns	p=0.5761
WT saline vs. DAT-KO rGF		ns	p=0.9823

Table S4. The statistical analyses of the number of return runs by DAT-KO and WT rats in the Hebb-Williams maze after saline, acute (aGF), and repeated (rGF) guanfacine administration. Two-way analysis of variance (two-way ANOVA) with Dunn's multiple comparisons post-hoc test was used. Statistically significant values indicated in green; ns – not significant.

Two-way ANOVA			P value
Interaction		ns	p=0.0901
treatment		*	p=0.0143
genotype		****	p<0.0001
Dunn's multiple comparisons post-hoc test			
	treatment		
WT vs. DAT-KO	saline	****	p<0.0001
WT vs. DAT-KO	aGF	ns	p=0.2182
WT vs. DAT-KO	rGF	ns	p=0.1226
	genotype		
saline vs. aGF	WT	ns	p>0.9999
saline vs. rGF	WT	ns	p=0.9960
saline vs. aGF	DAT-KO	**	p=0.0020
saline vs. rGF	DAT-KO	**	p=0.0089
compared to WT with saline administration			
WT saline vs. DAT-KO saline		****	p<0.0001
WT saline vs. DAT-KO aGF		ns	p=0.2393
WT saline vs. DAT-KO rGF		ns	p=0.1817

Table S5. The statistical analyses of pre-pulse inhibition index (PPI) for DAT-KO and WT rats after saline, acute (aGF), and repeated (rGF) guanfacine administration. Two-way analysis of variance (two-way ANOVA) with multiple comparisons Fisher's LSD post-hoc test was used. Statistically significant values indicated in green; ns – not significant.

Two-way ANOVA			P value
Interaction		ns	p=0.8395
treatment		**	p=0.0073
genotype		****	p<0.0001
multiple comparisons Fisher's LSD post-hoc test			
	treatment		
WT vs. DAT-KO	saline	**	p=0.0020
WT vs. DAT-KO	aGF	**	p=0.0075
WT vs. DAT-KO	rGF	**	p=0.0012
	genotype		
saline vs. aGF	WT	ns	p=0.1247
saline vs. rGF	WT	*	p=0.0305
saline vs. aGF	DAT-KO	*	p=0.0272
saline vs. rGF	DAT-KO	*	p=0.0485
compared to WT with saline administration			
WT saline vs. DAT-KO saline		**	p=0.0020
WT saline vs. DAT-KO aGF		ns	p= 0.2554
WT saline vs. DAT-KO rGF		ns	p=0.2288

Table S6. The statistical analyses of power spectral density of striatum LFP at various frequency bands in WT and DAT-KO rats. One-way analysis of variance (conventional one-way ANOVA for parametric and Kruskal-Wallis test for nonparametric data) was used. Post-test for linear trend demonstrated changes after saline, acute (aGF), and repeated (rGF) guanfacine administration; Dunn's multiple comparisons test was used to compare power spectra density after saline vs. aGF and after saline vs. rGF administration. Statistically significant values indicated in green; ns – not significant.

	WT		DAT-KO	
	Ordinary one-way ANOVA (parametric test)			
frequency band	P value	Post test for linear trend	P value	Sidak's multiple comparisons test<br>saline vs. aGF<br>saline vs. rGF
delta 0.9–3 Hz	ns; p=0.5226	ns; p=0.2580	**** p<0.0001	**** p<0.0001<br>** p=0.0027
	P value	Post test for linear trend	P value	Post test for linear trend
theta 4–8 Hz	**** p<0.0001	**** p<0.0001	**** p<0.0001	**** p<0.0001
alpha 9–11 Hz	*** p=0.0008	*** p=0.0004	**** p<0.0001	*** p=0.0006
	Kruskal-Wallis test (nonparametric test)			

	P value	Dunn's multiple comparisons test<br>saline vs. aGF<br>saline vs. rGF	P value	Dunn's multiple comparisons test<br>saline vs. aGF<br>saline vs. rGF
lower beta<br>12–19 Hz	** p=0.0046	* p=0.0216<br>** p=0.0044	**** p<0.0001	**** p<0.0001<br>**** p<0.0001
higher beta<br>20–29 Hz	** p=0.0030	** p=0.0046<br>** p=0.0089	**** p<0.0001	**** p<0.0001<br>**** p<0.0001
lower gamma<br>30–48 Hz	**** p<0.0001	**** p<0.0001<br>**** p<0.0001	**** p<0.0001	**** p<0.0001<br>**** p<0.0001
higher gamma<br>52–74 Hz	**** p<0.0001	**** p<0.0001<br>**** p<0.0001	**** p<0.0001	**** p<0.0001<br>**** p<0.0001

Table S7. The statistical analyses of power spectral density of prefrontal cortex LFP at various frequency bands in WT and DAT-KO rats. One-way analysis of variance (conventional one-way ANOVA for parametric and Kruskal-Wallis test for nonparametric data) was used. Post-test for linear trend demonstrated changes after saline, acute (aGF), and repeated (rGF) guanfacine administration; Dunn's multiple comparisons test was used to compare power spectra density after saline vs. aGF and after saline vs. rGF administration. Statistically significant values indicated in green; ns – not significant.

	WT		DAT-KO	
	Ordinary one-way ANOVA (parametric test)			
frequency band	P value	Post test for linear trend	P value	Post test for linear trend
delta<br>0.9–3 Hz	ns; p=0.3422	ns; p=0.4202	*** p=0.0003	**** p<0.0001
theta<br>4–8 Hz	**** p<0.0001	**** p<0.0001	ns; p=0.9540	ns; p=0.9422
	P value	Post test for linear trend	P value	Sidak's multiple comparisons test<br>saline vs. aGF<br>saline vs. rGF
alpha<br>9–11 Hz	**** p<0.0001	**** p<0.0001	**** p<0.0001	* p=0.0219<br>**** p<0.0001
	Kruskal-Wallis test (nonparametric test)			
	P value	Dunn's multiple comparisons test<br>saline vs. aGF<br>saline vs. rGF	P value	Dunn's multiple comparisons test<br>saline vs. aGF<br>saline vs. rGF
lower beta<br>12–19 Hz	**** p<0.0001	* p=0.0293<br>**** p<0.0001	**** p<0.0001	ns; p=0.1119<br>* p=0.0139
higher beta<br>20–29 Hz	**** p<0.0001	* p=0.0230<br>**** p<0.0001	* p=0.0261	ns; p=0.4216<br>* p=0.0140
lower gamma<br>30–48 Hz	**** p<0.0001	ns; p=0.3068<br>**** p<0.0001	**** p<0.0001	**** p<0.0001<br>ns; p=0.3938
higher gamma<br>52–74 Hz	**** p<0.0001	ns; p=0.1232<br>**** p<0.0001	**** p<0.0001	** p=0.0027<br>ns; p=0.2191

Table S8. The coherence between the activity of the prefrontal cortex (PFC) and Striatum (Str) at various frequency bands. One-way analysis of variance (ordinary one-way ANOVA) for coherence PFC-Str in WT and DAT-KO rats. Post-test for linear trend demonstrates changes after saline, acute (aGF), and repeated (rGF) guanfacine administration. Statistically significant values indicated in green; ns – not significant.

	WT		DAT-KO	
	Ordinary one-way ANOVA (parametric test)			
frequency band	P value	Post test for linear trend	P value	Post test for linear trend
delta 0.9–3 Hz	ns; p=0.6564	ns; p=0.8442	* p=0.0203	** p=0.0065
theta 4–8 Hz	**** p<0.0001	**** p<0.0001	**** p<0.0001	**** p<0.0001
alpha 9–11 Hz	**** p<0.0001	**** p<0.0001	**** p<0.0001	**** p<0.0001
lower beta 12–19 Hz	**** p<0.0001	**** p<0.0001	**** p<0.0001	**** p<0.0001
higher beta 20–29 Hz	ns; p=0.0720	ns; p=0.0531	**** p<0.0001	**** p<0.0001
lower gamma 30–48 Hz	**** p<0.0001	**** p<0.0001	**** p<0.0001	**** p<0.0001
higher gamma 52–74 Hz	**** p<0.0001	**** p<0.0001	**** p<0.0001	**** p<0.0001
